# Primary Closure Versus Delayed Primary Closure of Class III and IV Surgical Wounds Following Emergency Laparotomy: A Prospective Comparative Study

**DOI:** 10.7759/cureus.48965

**Published:** 2023-11-17

**Authors:** Rajalakshmi Venkateswaran, Shirish Bhagvat, Aishwarya Dutt, Harshal D Padekar, Najmeh Mirkhushal, Advaith A Chetan

**Affiliations:** 1 General Surgery, Grant Government Medical College, Mumbai, IND; 2 Critical Care Medicine, Chandramma Dayanand Sagar Institute of Medical Education and Research, Bangalore, IND

**Keywords:** negative pressure wound therapy, delayed primary closure, primary closure, surgical site infection, emergency abdominal surgery

## Abstract

Introduction

Emergency surgery has a high risk of complications due to the detrimental effect of perioperative sepsis and the relative lack of preoperative optimization of patients. Despite advances in critical care for the management of sepsis, its prevention is dependent on various patient and surgeon factors. Surgical site infection continues to be a major determinant of morbidity and mortality following emergency abdominal surgery, especially in contaminated or dirty wounds. This study aims to compare two techniques of abdominal wall closure, primary closure with subcutaneous suction drains and delayed primary closure following negative pressure wound therapy, in terms of incidence of surgical site infection and morbidity.

Materials and methods

The study was a prospective comparative study including 50 patients with an acute surgical abdomen requiring laparotomy. The patients were randomized into two groups, Group A (n=25) who underwent primary closure, and Group B (n=25) who underwent delayed primary closure. In Group B patients, a vacuum-assisted closure device was applied in the subcutaneous space for five days prior to the closure of the skin. Outcomes were compared in terms of the incidence of superficial and deep surgical site infection, its association with diabetes mellitus, and the total duration of hospital stay. A chi-square test and an unpaired t-test were used for the test of significance.

Results

A total of 50 patients, comparable in age, were included in the study. The overall incidence of surgical site infection was significantly higher in patients of Group A as compared to Group B (p=0.0046). There was a positive correlation between diabetes mellitus and the occurrence of wound infection in both groups with the odds ratio being 2.67 and 2.38 respectively. The incidence of superficial wound infection was significantly higher in Group A when compared to Group B (52% versus 24%; p=0.04). Deep surgical site infection was higher in patients of Group A (20% versus 8%) but was not statistically significant (p=0.22). The average duration of hospital stay was 41.56 ± 6.96 and 37.86 ± 6.68 days for patients who developed complications from Groups A and B respectively, while it was nearly two and a half times lower in uncomplicated cases of Groups A and B (11.71± 1.70 days and 16.58± 1.06 days respectively). The one-tailed unpaired t-test showed a significant difference in means of hospital stay between patients with and without complications (T: 17.06, critical value: 1.677).

Conclusion

Delayed primary closure is an effective method of managing contaminated and dirty wounds following emergency laparotomy. Negative pressure wound therapy is one technique for preventing wound bed infection and accelerating wound healing in such cases. By combining the above in emergency surgeries, the incidence of surgical site infection and duration of hospital stay can be significantly reduced.

## Introduction

The term "acute abdomen" refers to a spectrum of medical and surgical conditions leading to a sudden onset, progressive abdominal pain that requires hospitalization and urgent intervention. The most common surgical causes of an acute abdomen include acute appendicitis, cholecystitis, renal colic, and peritonitis due to intestinal perforation, intestinal obstruction, and bowel ischemia [1]. In cases requiring emergency laparotomy, the incidence of postoperative surgical site infection (SSI) leading to wound failure is quite high, at around 30-40% [2].

The Centre for Disease Control and Prevention (CDC) has classified surgical wounds into four types based on the level of contamination in the operative field, wherein class I: clean; class II: clean contaminated; class III: contaminated; and class IV: dirty [3]. Class III refers to open, fresh accidental wounds or conditions with gross uncontrolled spillage from the gastrointestinal tract with acute non-purulent inflammation. Class IV refers to traumatic wounds with devitalized tissue wherein the organism responsible for infection is present in the field pre-operatively [3]. The risk of postoperative SSI in Class III and IV surgical wounds is around 30-35% and 40-50%, respectively [4].

The CDC has further categorized SSIs as superficial, deep, and organ-space SSIs [3]. Superficial SSI refers to a breach in skin and subcutaneous tissue while deep SSI refers to a condition where there is dehiscence of the fascial layers [3]. In the present study, superficial SSIs implied wound gaping while deep SSIs implied a burst abdomen. This leads to high morbidity and mortality among patients [4]. SSI management includes antibiotics, serial wound debridement, daily dressings, and lengthy in-hospital care. The proven efficacy of negative pressure therapy on exudative wounds [5] has been explored in this study for managing abdominal wounds. The present study compares the efficacy of the two techniques, primary closure and delayed primary closure of the skin and subcutaneous tissue following emergency abdominal surgery (EAS) in terms of the occurrence of SSI.

## Materials and methods

This study was a prospective comparative study conducted in a single tertiary care center in Mumbai from February 2021 to February 2022 after obtaining approval from the Institutional Ethics Committee (IEC/PG/ 322/Feb/2021). A total of 50 patients between the ages of 18-65 years who were diagnosed with an acute surgical abdomen and expected to have a CDC class III/IV surgical wound post laparotomy such as those with hollow viscus perforation, penetrating abdominal trauma, or bowel ischemia were included in this study. Pregnant patients were excluded from the study. 

All patients who were part of the study were operated by the same surgeon. Two types of abdominal wall closure techniques were employed in the study, primary closure (PC) and delayed primary closure (DPC). Group A (n=25) included the patients who underwent primary closure of the abdominal wall while Group B (n=25) included those who underwent delayed primary closure. Written and informed consent were taken from all patients. Patients were randomized into these two groups by simple unstratified random sampling using a pre-determined computer-generated randomization chart.

In the postoperative period, besides demographic data, comorbidities, the incidence of superficial SSI, deep SSI, and hospital stays amongst the two groups were documented. All patients were discharged only when they had a healthy scar after suture removal. 

Abdominal wall closure techniques

Primary Closure of Abdominal Wall

**Figure 1:**
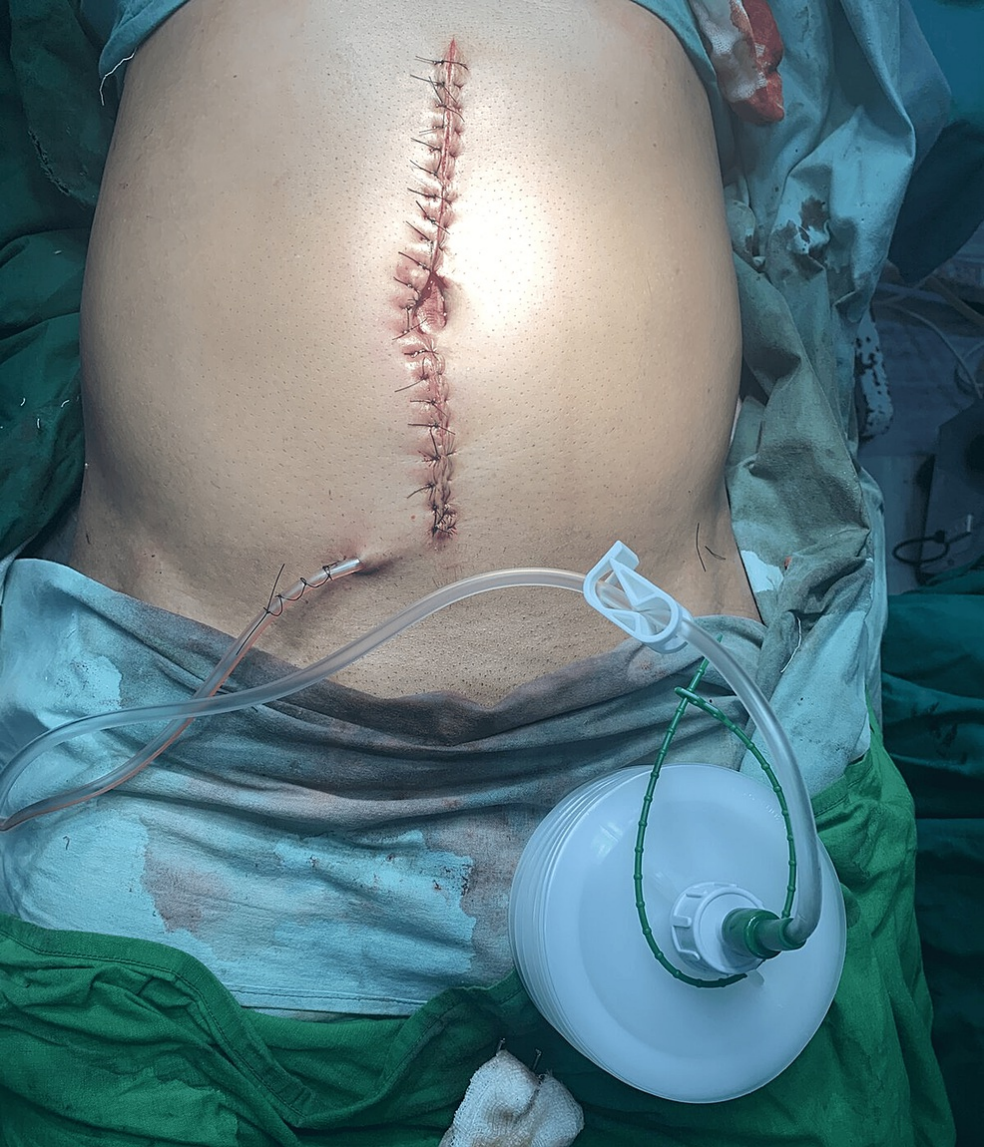
Primary closure of the abdomen wall with SSD. SSD: subcutaneous suction drain

The edges of the linea alba were approximated by taking simple interrupted sutures with polydioxanone suture 2-0. A subcutaneous suction drain (SSD) was placed in the subcutaneous plane and the overlying skin was approximated using nylon sutures in an interrupted manner (Figure 1).

The SSD was removed on POD (post-operative day) 8. In cases with pus discharge from the suture line needing removal of sutures, the SSD was removed earlier.

Delayed Primary Closure of Abdominal Wall

**Figure 2:**
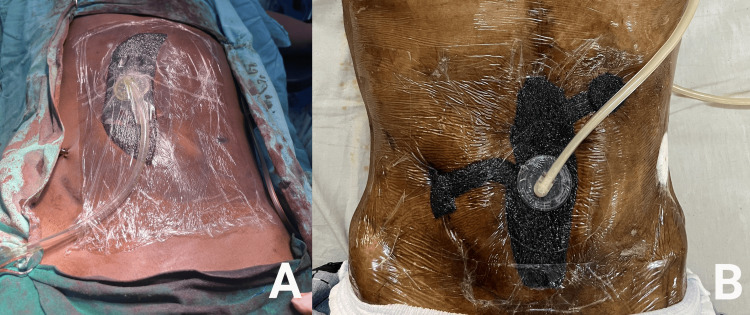
NPWT over the wound bed. 2A: VAC apparatus placed over rectus sheath following EAS with a secured intraabdominal drain. 2B: VAC apparatus placed in the subcutaneous space following laparotomy for abdominal stab injury NPWT: negative pressure wound therapy; VAC: vacuum-assisted closure; EAS: emergency abdominal surgery

The edges of the Linea alba were approximated using Polydioxanone suture, simple interrupted sutures were taken. The vacuum-assisted closure (VAC) device was applied and the procedure was followed as per these steps (Figure 2): first, a microporous foam of appropriate size was placed over the rectus sheath; second, a perforated drain tube was placed into the foam; third, a transparent adhesive membrane was placed covering the foam, the tube, and 6-7cm of healthy skin to form an airtight and watertight seal; fourth, the distal end of the tube was connected to a vacuum pump.

An intermittent negative pressure cycle was maintained keeping pressures between -70mmHg to -85mmHg. If the output was serosanguinous, the VAC apparatus was removed on POD 5, and an approximation of the skin edges was done by taking interrupted vertical mattress sutures using nylon under local anesthesia. If pus was visualized in the apparatus, the VAC apparatus was removed earlier.

In both situations, if there was an SSI, patients were managed with daily dressings till the wound was healthy to undergo secondary suturing.

Statistical analysis

The data was collected and entered into Microsoft Excel and analyzed using Python 3. The data was presented as percentages and categories and then presented as tables. The chi-square test and the one-tailed unpaired t-test were used for comparison of variables and test of significance. A value of less than 0.05 from the chi-square test and an absolute value of the calculated t more than the critical value from the t-test was considered statistically significant.

## Results

**Figure 3:**
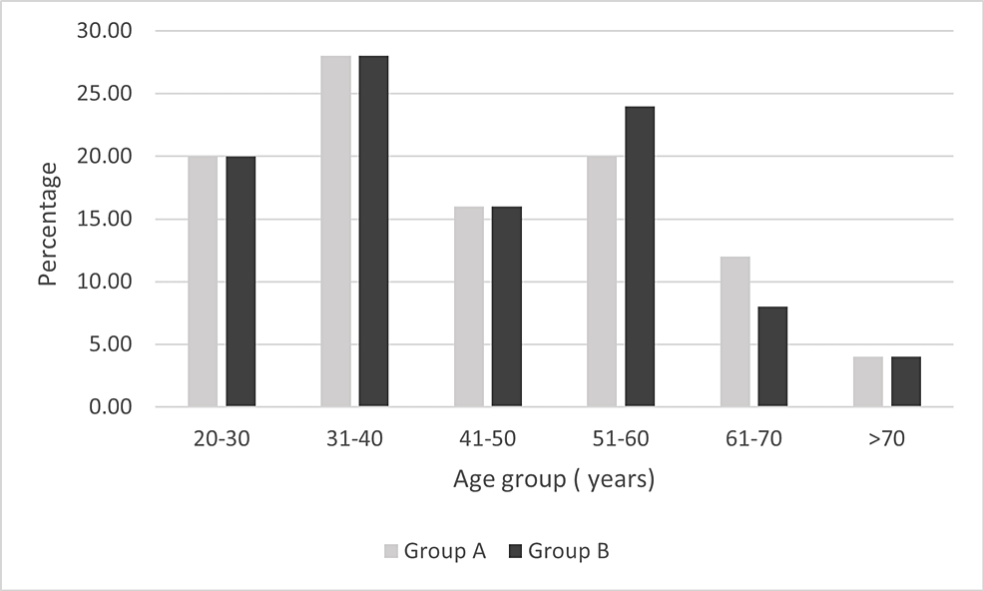
Age-wise distribution of the study subjects

On comparing the 50 patients included in the study, the age-wise distribution between the two groups was comparable (Figure 3).

The overall incidence of SSI was more in Group A as compared to Group B (18 versus eight), which was statistically significant when compared using the chi-square test (p-value=0.0046).

Out of the total patients in each group, 15 patients of Group A and 12 patients of Group B had diabetes mellitus (DM). On analysis, there was a positive association of DM with SSI in both groups with the odds ratio being 2.67 and 2.38 respectively.

**Figure 4:**
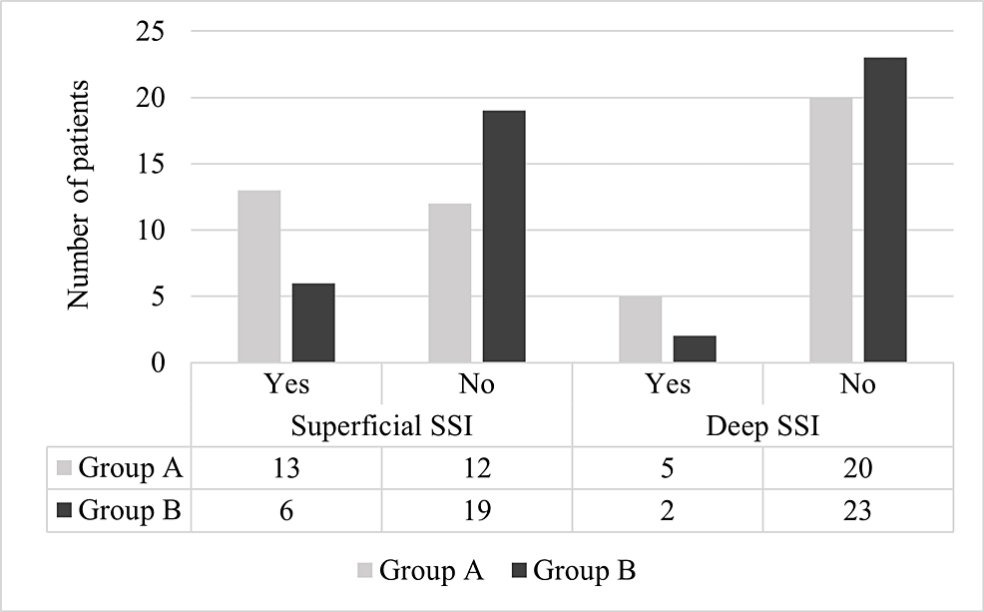
Comparative analysis regarding the incidence of superficial and deep SSI among the two groups SSI: surgical site infection The chi-square test showed a significantly higher incidence of superficial SSI in Group A as compared to Group B with a p-value of 0.041.

The individual incidences of superficial and deep SSI are given in Figure 4. Comparative analysis using a chi-square test revealed a statistically significant difference in the incidence of superficial SSI between Group A and Group B, with the p-value being 0.041, and a non-significant incidence of deep SSI amongst the two groups (p-value=0.22).

**Figure 5:**
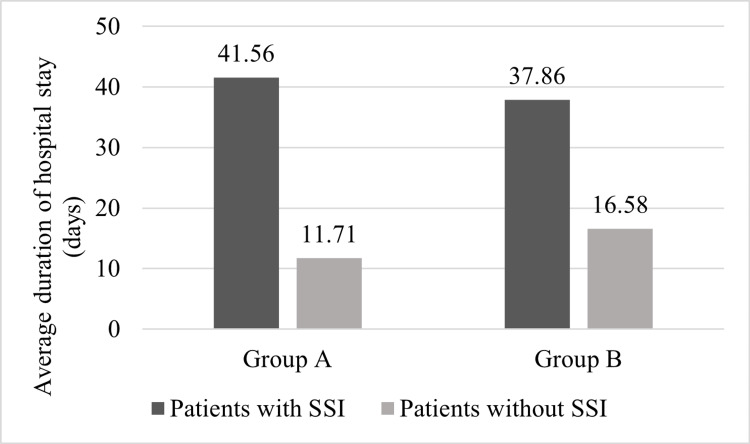
Comparison of duration of hospital stays SSI: surgical site infection The unpaired t-test revealed a significant difference between the means of duration of hospital stay in patients with and without complications (T: 17.0619, critical value: 1.677).

The average duration of hospital stays for patients of Group B who did not develop SSI was 16.58 ±1.06 days whereas it was nearly two and a half times more for patients of Group A who developed SSI, at 41.56 ± 6.96 days (Figure 5). However, the least duration of hospital stay (11.71 ± 1.70 days) was amongst the Group A patients who had an uneventful postoperative course. The one-tailed unpaired t-test test showed a significant difference between the means of hospital stays among patients with and without wound complications (T: 17.06; degree of freedom: 48; critical value: 1.677).

## Discussion

Emergency abdominal surgery (EAS) remains a challenge to many surgeons due to the multitude of intra-operative and postoperative complications that accompany these procedures [6]. The most common and detrimental complication is SSIs, which may result in septic shock, admissions to the ICU, repeated surgeries, and death.

Studies have shown that there is a significant association between the incidence of SSI and CDC class III or IV surgical wounds [4,7]. Important factors responsible for causing these are the presence of infected interstitial fluid in the wound bed, poor nutritional status, and immunocompromised state. A systemic review published in 2015 has shown diabetes to be an independent risk factor for causing SSIs, with an odds ratio of 1.53 [8]. Uncontrolled diabetes [7], anemia [9], low albumin, etc. undoubtedly have an adverse effect on wound healing, but it is impossible to correct them pre-operatively as patients with secondary peritonitis require EAS. Hence, appropriate interventions by surgeons are needed to prevent infection and enhance wound healing in these cases.

The type of abdominal wall closure is one method that can be modified by surgeons to prevent SSIs. In this regard, negative pressure wound therapy (NPWT) significantly reduces the incidence of SSIs [10] and promotes wound healing, simultaneously providing time for the body’s immune system to fight sepsis and the inflammatory response to subside. It causes wound size shrinkage and brings the wound edges together by exerting tension forces on it [11]. Of particular concern is the fluid collecting in the subcutaneous space, which acts as a nidus for bacterial growth and causes tension on the suture line. NPWT, by constantly evacuating this fluid prevents infection, removes local inflammatory mediators, and relieves suture line tension. The other significant effect is enhanced granulation tissue formation due to increased cellular proliferation and hypoxia-induced angiogenesis [11].

NPWT also maintains fascial integrity by decreasing tension on the rectus sheath edges, thus minimizing the chance of a burst abdomen. A meta-analysis conducted by Meyer and colleagues in 2021 showed that the rates of SSI amongst patients who were managed with prophylactic NPWT over closed abdominal incisions were significantly less as compared to the patients managed conventionally (p-value = 0.009) [12]. The same ideology was used in this study but with modifications on the plane of placement of the VAC apparatus.

Studies have shown that SSDs in EAS reduce SSI and wound dehiscence [13-15]. However, its beneficial role in CDC class III / IV wounds is not specifically highlighted. Also, in most studies, the SSDs were removed on POD 3 [13] or when the output was less than 5ml/ 24 hours [14]. An analysis of 1400 patients published in 2018 showed that the most common period of occurrence of SSI is 5-15 days [16]. Moreover, blockage and loss of suction pressure are two disadvantages of using SSDs. Despite retaining SSDs, 17 of 25 patients of Group A in the present study developed SSI, showing that they are of minimal benefit in contaminated and dirty wounds.

The benefits of NPWT over SSDs are that the intermittent negative pressure cycle provided by a VAC apparatus has a stronger suction force as compared to that provided by the spring-loaded device. Once the pressure drops in an SSD, its function ceases until a manual effort is taken to re-instill the suction force. While it remains discharged, its function of evacuating the extracellular fluid is lost. This disadvantage is not seen in the VAC apparatus as the suction pressure is maintained between the desired negative range continuously due to it being run by electric power. Pressures can be raised up to -100 mmHg in heavily exudating abdominal wounds, which cannot be achieved with SSDs. Apart from preventing fluid accumulation, NPWT also stimulates the wound healing process, which is of utmost benefit in immunocompromised and septic patients.

SSIs significantly prolong hospital stays and increase the financial burden of the affected patients [10,17,18], as was seen in the present study. In cases with uneventful post-operative courses, the two disadvantages of DPC are a slightly increased hospital stay (approximately five to seven days) and the requirement for a repeated surgical procedure. However, the benefit of a significantly lower incidence of SSI outweighs the drawbacks. 

The limitation of this study is the small study population comprising only 50 subjects. Multicentric studies and larger trials are required for better validation of this technique.

## Conclusions

EAS is associated with high rates of SSIs, especially in CDC class III and IV surgical wounds resulting in prolonged hospital stays and repeated surgeries. Diabetes mellitus is a major contributing factor in the development of SSIs.

NPWT is beneficial in the management of infected wounds as it promotes granulation tissue growth apart from removing the stagnant edematous fluid. Based on the results of our study, we conclude that, in patients with contaminated or dirty laparotomy wounds, application of NPWT over the closed rectus sheath (subcutaneous space) for a period of five days prior to skin closure ie., delayed primary closure can significantly reduce the rates of superficial and deep SSIs, facilitating early discharge from the hospital.
